# Commentary on: “Focal cryosurgical ablation of the prostate: A single institute’s perspective”

**DOI:** 10.1186/1471-2490-13-39

**Published:** 2013-08-02

**Authors:** Adam R Metwalli, Peter A Pinto

**Affiliations:** 1 Urologic Oncology Branch, National Cancer Institute, NIH, Building 10, Room 2W-5940, 10 Center Drive, MSC 1210, Bethesda, MD 2089s, USA

**Keywords:** Prostate cancer, Therapy, Focal, Cryoablation, Image guided surgery

## Abstract

The morbidity of whole gland treatment for prostate cancer is significant. Given the low risk of prostate cancer specific mortality for most men diagnosed with prostate cancer, alternative therapies such as sub-total or hemi-ablation of the prostate and focal ablation of prostate tumors are being investigated. The developing role of imaging for prostate tumors will dramatically change and likely improve the treatment morbidity for low risk prostate tumors. Commentary on: http://www.biomedcentral.com/1471-2490/13/2.

## Commentary

The concept of focal therapy for prostate cancer is a developing area in urologic oncology and has proven a fertile ground for ongoing investigation. The authors present their very early results on what they describe as “focal” therapy of the prostate using cryoablative techniques [1]. They note that while oncologic data is not yet available, the procedure is tolerable and appears to result in acceptable biochemical outcomes. Nevertheless, in their conclusions they note that “the true extent of cancer control remains in question….” We feel this statement really epitomizes the challenge of applying sub-total ablative therapies to prostate cancer at this time. In truth, the issues are twofold: 1) adequately identifying the extent of the cancer within the gland is imperative to allow reasonable certainty about cancer control after treatment and 2) truly focal therapy cannot be applied without utilization of imaging modalities and so the term “focal” therapy is actually a misnomer for hemi-ablative and subtotal ablative approaches.

With respect to the first issue, the authors used a 3D mapping ultrasound guided transperineal saturation biopsy to determine the extent of the cancer. The limitations of ultrasound in identifying tumors within the prostate are well documented [2,3]. So this approach attempts to systematically sample regions of the prostate, but it does not totally obviate the concern that clinically relevant tumors may be missed. Thus, it can be argued that adequate focal therapy cannot be performed in the absence of image-guided targeted biopsies since the extent of disease cannot be as accurately determined. These techniques are still in development [4-6] and so the degree of uncertainty about what is actually being ablated during focal ablation in the absence of imaging is a real concern.

The corollary to the concern about the extent of disease without adequate imaging is the efficacy of therapy when the ablation is not image-guided. If a practitioner cannot identify the tumor to be treated, then true focal ablation is impossible. One could argue it is tantamount to the difference between a partial nephrectomy for a small renal mass and a hemi-nephrectomy for the same mass.

No doubt, these data show that the morbidity of the therapy is reasonable and certainly less than whole gland extirpation based on historical controls. This manuscript adds to the growing data that hemi-ablation and subtotal ablation of the prostate are well tolerated and feasible [7-9]. The authors are to be commended for their efforts to add to the growing knowledge in this field.

Finally, the question remains about the necessity to treat disease that is highly unlikely to have a negative impact during the lifetime of the patient. Data such as these beg the question about whether very low risk prostate cancer should be treated at all. As these data suggest, prostate hemi-ablation appears to be preferable, at least in the short term, to whole gland ablation with respect to morbidity and quality of life. But these questions really emphasize the need for a change of mindset not only for treating physicians, but more importantly for the patients, who must become comfortable with the concept and execution of active surveillance for low risk prostate cancer. To the author’s credit, these data add to the literature that should a patient progress while on active surveillance, reasonable alternatives to radical surgery or radiotherapy are being developed.

## Competing interests

The authors declare that they have no competing interests.

## Authors’ contributions

ARM: conception, writing. PAP: critical review. Both authors read and approved the final manuscript.

## Authors’ information

First author: Adam R Metwalli.

Second author: Peter A Pinto.

## Pre-publication history

The pre-publication history for this paper can be accessed here:

http://www.biomedcentral.com/1471-2490/13/39/prepub
